# Supplementary material for: Levels, Distributions, and Potential Risks of Hexachlorobutadiene from Two Tetrachloroethylene Factories in China
Source: Int J Environ Res Public Health. 2023 Mar 14;20(6):5107. doi: 10.3390/ijerph20065107 (PMC10048807; doi:10.3390/ijerph20065107)
Supplement: Supplementary file 1 [file ijerph-20-05107-s001.zip › ijerph-2233675-supplementary.pdf]

SUPPLEMENTARY DATA

for

Levels, distributions, and potential risks of hexachlorobutadiene from two tetrachloroethylene factories in China

Content

Table S1..1

POPs Toolkit:..1

Table S1 Input Parameters for assessment of HCBD exposure and health risk.

Exposure parameter	meaning	Adults
IR_S (kg/day)	Accidental soil ingestion rate for adult	0.00002
AF_{GIT}	Absorption Factor for the gastrointestinal tract	1
D_{Hour}(hour/day)	Hours per-day with exposure (0 - 16)	8
D_{Days}(day/week)	Days in a week with exposure (0 - 7)	5
D_{weeks}(week/year)	Weeks in a year with exposure (0 - 52)	50
P_{Air}	Concentration of particles in the air.	87.5
BW (kg)	Body Weight of Receptor	71
IR_A (kg/day)	Inhalation rate	0.66
AF_{Inh}	Absorption Factor for the lungs	1
SA_H (cm^2)	Surface area of hands	3390
SL_H (kg/ cm^2-event)	Soil loading to exposed skin	0.00001
AF_{Skin}	Absorption Factor for the skin	0.1
EF(event/day)	number of dermal exposures per day	2
TDI (mg/ kg-day)	Tolerable daily intake	0.0002

POPs Toolkit:

Accidental Soil Ingestion Dose Calculation:

$$Dose_{SoilIngestion} = \frac{(C_s \times IR_s \times AF_{GIT} \times D_{Hours} \times D_{Days} \times D_{Weeks} \times D_{Years})}{BW \times 16 \times 365 \times LE}$$

Dermal contact with contaminated soil Dose Calculation:

$$Dose_{DermalContact} = \frac{(C_s \times SA_H \times SL_H \times AF_{Skin} \times EF \times D_{Days} \times D_{Weeks} \times D_{Years})}{BW \times 365 \times LE}$$

Inhalation of contaminated particles Dose Calculation:

$$Dose_{ParticleInhalation} = \frac{(C_s \times P_{Air} \times IR_A \times AF_{Inh} \times D_{Hours} \times D_{Days} \times D_{Weeks} \times D_{Years})}{BW \times 365 \times LE \times 10e^9}$$

Calculation of Hazard Quotient:

$$HQ = \frac{(Dose_{SoilIngestion} + Dose_{ParticleInhalation} + Dose_{DermalContact})}{TDI}$$
